# Rise of the BQ.1.1.37 SARS-CoV-2 Sublineage, Italy

**DOI:** 10.3390/diagnostics13051000

**Published:** 2023-03-06

**Authors:** Daniele Focosi, Pietro Giorgio Spezia, Anna-Lisa Capria, Federico Gueli, Scott McConnell, Federica Novazzi, Mauro Pistello

**Affiliations:** 1 North-Western Tuscany Blood Bank, Pisa University Hospital, 56124 Pisa, Italy; 2 Division of Virology, Pisa University Hospital, 56124 Pisa, Italy; 3 Independent Researcher, 2210 Como, Italy; 4 Department of Molecular Microbiology and Immunology, Johns Hopkins Bloomberg School of Public Health, Baltimore, MD 21205, USA; 5 Laboratory of Microbiology, ASST Sette Laghi, 21100 Varese, Italy; 6 Retrovirus Center and Virology Section, Department of Translational Research, University of Pisa, 56100 Pisa, Italy

**Keywords:** SARS-CoV-2, COVID-19, BQ.1.1.37, Cerberus, Italy

## Abstract

BQ.1.1 has dominated the Europe and Americas COVID-19 wave across the 2022–2023 winter, and further viral evolution is expected to escape the consolidating immune responses. We report here the emergence of the BQ.1.1.37 variant in Italy, peaking in January 2022 before suffering competition by XBB.1.*. We attempted to correlate the potential fitness of BQ.1.1.37 with a unique two-amino acid insertion within the Spike protein.

The BA.5-derived BQ.1.1 Omicron sublineage has been the eighth SARS-CoV-2 sublineage to achieve global absolute dominance (>50% of all sequences in a given week) during the ongoing COVID-19 pandemic. Its dominance has so far been restricted to Europe and the Americas, with increased circulation facilitating the emergence of descendant sublineages. As of 24 February 2023, 60 BQ.1.1 sublineages have been designated by PANGOLIN [1], some of them requiring further aliases (CZ, DU, DN, DK, DP, CW, DM, DT).

We report here on the emergence of the recently (23 January) designated BQ.1.1.37 [2], where the defining nucleotide mutation is T1453C (following A17039G (ORF1b:N1191S)), and the most interesting feature is a unique two-amino acid (AE) insertion at the indel hotspot in the fifth loop (N5) of the N-terminal domain (NTD) of the Spike protein (S:Y248D ins_S:247:SAE).

On 25 February 2023, we retrieved from GISAID [3,4] and CoV-Spectrum database [5] the exact same set of 215BQ.1.1.37 sequences. Notably, the query for the GISAID database (Spike_ins248AED + Spike_Y248S) is slightly different from the one for the CoV-Spectrum database (S:Y248D + ins_S:247:SAE), the latter having an average 5-day lag from GISAID. The sequences are available as EPI_SET_230225ku at the link https://doi.org/10.55876/gis8.230225ku (accessed on 25 February 2023). We generated a phylogenetic tree (Supplementary Figure S1) using Molecular Evolutionary Genetic Analysis (MEGA) software v.1.11.09 (https://megasoftware.net/ (accessed on 25 February 2023)); the Maximum Composite Likelihood method was used to compute evolutionary distances, expressed as the number of base substitutions per site. The isolates that were sequenced in our laboratories were labeled in red and the tree was edited using the interactive Tree of Life (iTOL) tool v.6 (https://itol.embl.de/ (accessed on 25 February 2023)). The first sequence appeared in Lombardy on 15 November 2022. More than half of those sequences (135) were reported from Italy (mostly from Emilia-Romagna, Umbria and Lombardy regions), with the incidence rate reaching 4.8% on 6 February. A total of 67 more sequences were deposited from other European countries (Germany 13, Spain 11, Austria 8, Netherlands 7, Sweden 6, Belgium 5, Ireland 4, France 3, Denmark 3, Portugal 2, Luxembourg 2, Poland 1, Switzerland 1, Czechia 1), and 13 more from other continents. Eventually, because of partial labeling in metadata, we could not document whether some of the cases outside Italy had been locally imported from Italy.

Of interest, in the past 4 months BQ.1.1.37 has a current relative growth advantage across Europe of 38% (CI 32–44%) (Figure 1). As of 26 January 2023, CoV-Spectrum collection #24 analysis, maintained by one of the authors [6], showed that BQ.1.1.37 was the fourth fastest growing sublineage worldwide compared to an S:F486P baseline, but the arrival of the wave led by the much fitter XBB.* lineages moved BQ.1.1.37 to the 25th rank on 25 February 2023; hence, BQ.1.1.37 is likely to be outcompeted soon (e.g., XBB.1.5 having a relative growth advantage of 19% and XBB.1.9 of 39% over BQ.1.1.37).

BQ.1.* was dominating Italy as of January 2023 (BQ.1.1 22%, BQ.1.22 13.5%, BQ.1 5.8%). Italy has a sequencing rate much less than 1% of positive samples, making the occurrence of the BQ.1.1.37 peak highly significant. Furthermore, the growth of BQ.1.1.37 partly continued in Italy even after the arrival of the XBB.1.5 (Figure 2).

BQ.1.1.* sublineages are already baseline resistant to the RBD-binding monoclonal antibodies (mAb) cilgavimab, tixagevimab, sotrovimab and bebtelovimab. Figure 3 shows that the two-amino acid insertion is unlikely to further disrupt the receptor binding site (RBD) of the Spike protein, but can instead cause immune escape to NTD-directed mAbs according to the classification by Finkelstein et al. [7], i.e., those that prevent conformational changes necessary for fusion or cause steric interference. For example, Andreano et al. reported that an 11-amino acid insertion between Y248 and L249 emerging after 13 passages in the presence of convalescent serum caused total lack of neutralization [8].

Genome insertions play a relevant role in the evolution of coronaviruses in general [9], and of SARS-CoV-2 in particular [10]. A 12-nt insert was at the origin of SARS-CoV-2, generating a second furin-cleavage site (FCS). Several lineages contain insertions in their Spike proteins, e.g., A.2.5 and B.1.214.2 variants which both have insertions in the aa~210 insertion hotspot region [11], VOI Mu (YY144-145TSN, contributing to immune resistance [12]), VOC Omicron BA.1 (ins214EPE), and several sublineages of Omicron BA.2 [13] such as BA.2.52 (ins_S:247:SGE). Among the BA.2-paraphyletic BA.4/5 lineages, XBB.1.8 has ins_S:186:SGG, and BS.1 has ins_S:212:NGE.

Interestingly, five out of the fastest BQ.1.1 sublineages so far have one or two mutations/insertions at 247/248, namely: EH.1 (with S:S247N and S:Y248S, which is now the 14th fastest lineage in the world), BQ.1.1.45 (with S:Y248D, which is now the 21th fastest lineage), EA.1 (with ins_S:247:SKWL, which is the 24th fastest lineage), BQ.1.1.37 (the 25th fastest lineage worldwide) and BQ.1.1.63 (S:Y248H). Another BQ.1.* sublineage, BQ.1.28, harbors a different insertion in the Spike protein, namely ins248RWMD. Remarking on the importance of the residues 247 and 248, in Spring 2022 in India, in the middle of the BA.2.75 and BA.5 wave, only two sublineages were able to compete, notably BA.2.76 (S:Y248N, also causing a notable outbreak in China [14]) and BA.2.38.1 (S:S247N + S:Y248S).

Most insertions cluster in the Spike NTD and at the S1/S2 cleavage site: while many insertion sequences appear to be viral in origin, a subset of insertions show homology to RNA sequences from host transcripts, implying incorporation of short host RNA sequences during viral genome replication [15]. Analysis of homology of Omicron ins214EPE and flanking regions suggests that the template switching event could have involved the genomes of SARS-CoV-2 variants (e.g., B.1.1 strain), other human coronaviruses that infect the same host cells as SARS-CoV-2 (e.g., HCoV-OC43 or HCoV-229E), or a human transcript expressed in a host cell that was infected by the Omicron precursor [16].

Despite the fact that the growth rates of BQ.1.1.37 are likely to be tapered along the XBB.1.* wave, BQ.1.1.37 has represented a clear example of a geographically restricted diversification and of the impact of Spike deletions on growth rates.

**Figure 1:**
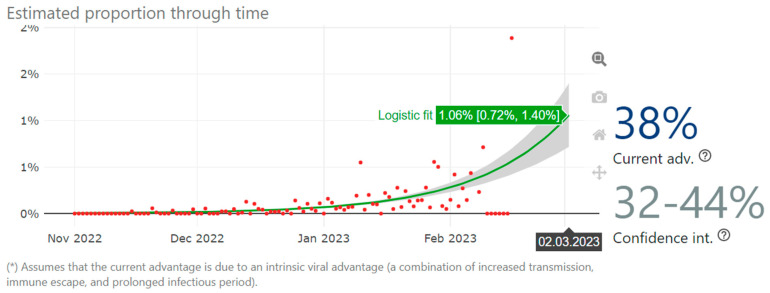
Relative growth advantage of BQ.1.1.37 in Europe since 1 November 2022 to 25 February 2023, calculated using CoV-Spectrum.org over a S:F486P baseline [5]. The model assumes that the increase or decrease of the proportion of a variant follows a logistic function. It fits a logistic model to the data by optimizing the maximum likelihood to obtain the logistic growth rate in units per day [17].

**Figure 2:**
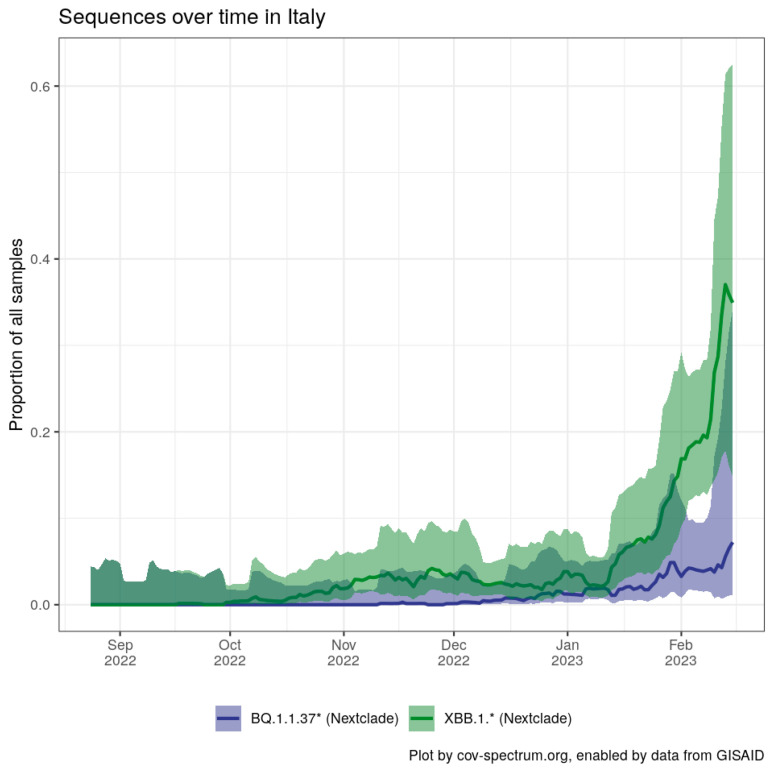
Comparison of BQ.1.1.37 and XBB.1.5 expansions in Italy. Chart generated using CoV-Spectrum [5].

**Figure 3:**
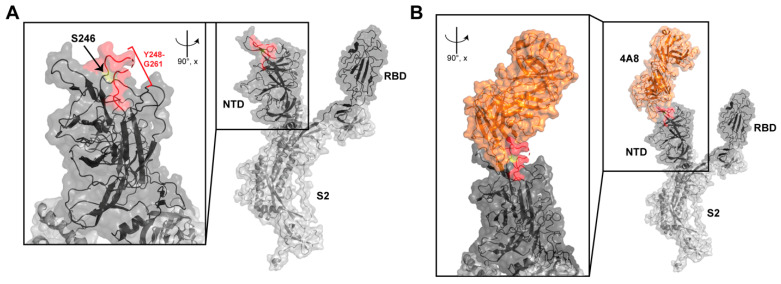
Localization of the BQ.1.1.37 NTD insertion with respect to a neutralizing antibody specific to the NTD. All three-dimensional molecular representations were generated with PyMOL 2.5.2 (Schrodinger). (**A**) SARS-CoV-2 S protein is displayed as cartoon representation overlaid on the space-filling surface. RBD, NTD and S2 are colored as black, dark grey and light grey, respectively. The position of the insertion at position S246, and residues C-terminal to that position in the loop are highlighted in yellow and red, respectively. (**A**, inset) An expanded view of the NTD, rotated 90 degrees about the *x*-axis is displayed to visualize the loop residues that would be displaced by the insertion in the BQ.1.1.37 S protein variant. (**B**) The structure of the complex between NTD and nAb 4A8 is displayed as before with the 4A8 Fab structure colored orange. (**B**, inset) An expanded view of the nAb interaction, rotated 90 degrees about the *x*-axis is displayed to highlight the probable perturbation of the 4A8 epitope caused by the S246 insertion in NTD.

## Data Availability

This manuscript did not generate any dataset.

## Supplementary Materials

The following supporting information can be downloaded at: https://www.mdpi.com/article/10.3390/diagnostics13051000/s1.
