# Supplementary figures and images for: Rise of the BQ.1.1.37 SARS-CoV-2 Sublineage, Italy
Source: Diagnostics (Basel). 2023 Mar 6;13(5):1000. doi: 10.3390/diagnostics13051000 (PMC10001149; doi:10.3390/diagnostics13051000)

Tree scale: 0.0001

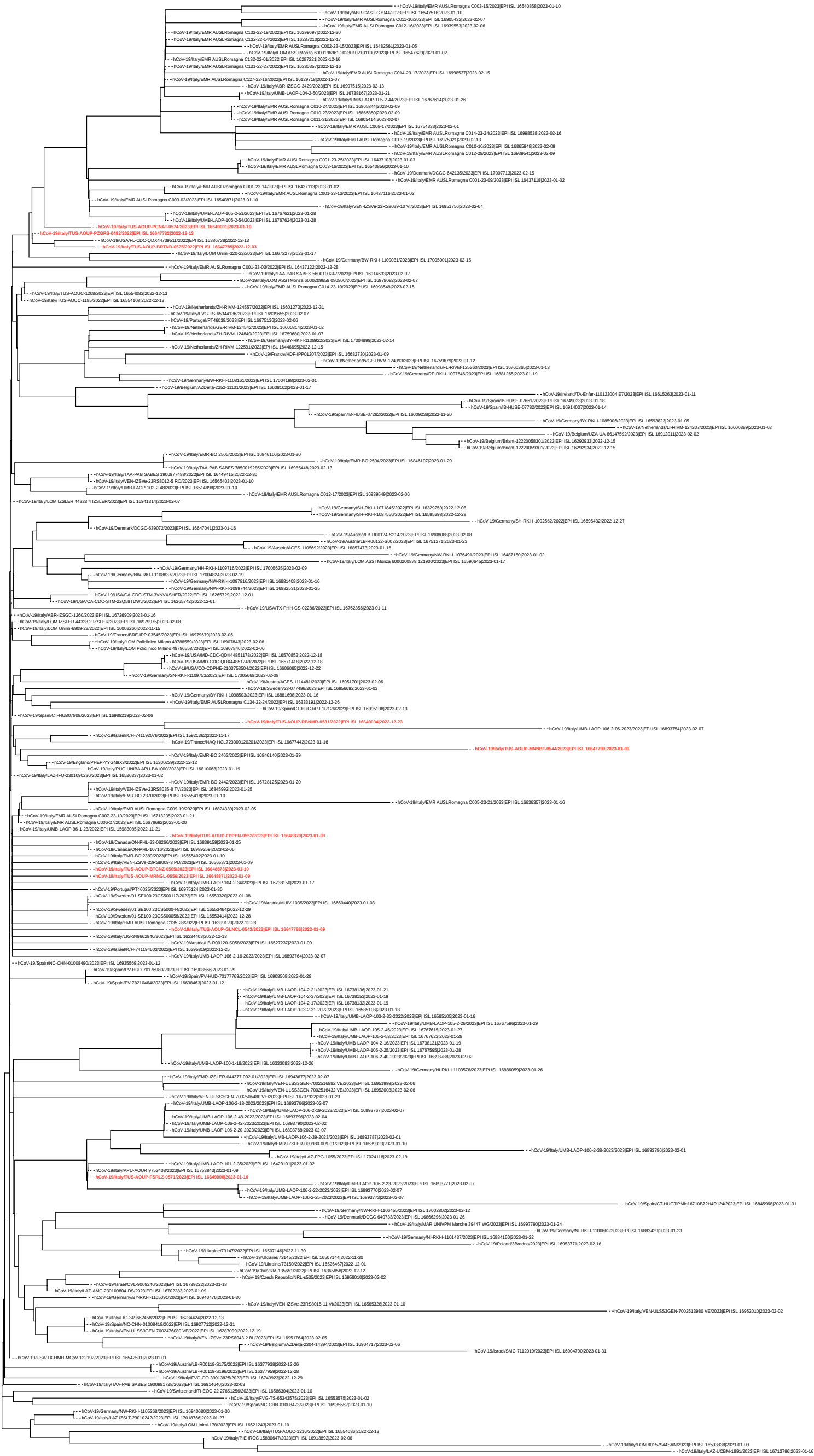

Supplement: Supplementary file 1 [file diagnostics-13-01000-s001.zip › diagnostics-2214337-supplementary.pdf]
